# The prenatal video‐feedback intervention to promote positive parenting for expectant fathers (VIPP‐PRE): Two case studies

**DOI:** 10.1002/imhj.22006

**Published:** 2022-08-01

**Authors:** Noor de Waal, Kim Alyousefi‐van Dijk, Renate S. M. Buisman, Martine W. F. T. Verhees, Marian J. Bakermans‐Kranenburg

**Affiliations:** ^1^ Clinical Child & Family Studies Faculty of Behavioral and Movement Sciences Vrije Universiteit Amsterdam The Netherlands; ^2^ Forensic Family and Youth Care Studies Faculty of Social and Behavioral Sciences Leiden University Leiden The Netherlands; ^3^ Quantitative Psychology and Individual Differences KU Leuven Leuven Belgium

**Keywords:** father, intervention, involvement, prenatal, sensitivity, video‐feedback, prenatal, padre, información de video, intervención, sensibilidad, Mots clés: Prénatal, Père, Rendu‐vidéo, Intervention, Sensibilité, Intervention, Engagement, Pränatal, Vater, Video‐Feedback, Intervention, Sensibilität, 出生前、父親、ビデオフィードバック、介入、感受性, 关键词: 产前, 父亲, 视频反馈, 干预, 敏感性, **الكلمات الرئيسية**: قبل الولادة ، الأب ، التغذية الراجعة بالفيديو ، التدخل ، الحساسية

## Abstract

Although parenting interventions including expectant fathers are scarce, they yield promising results. The Prenatal Video‐feedback Intervention to promote Positive Parenting (VIPP‐PRE) is a recently developed intervention, that is both manualized and personalized, aiming to enhance paternal sensitivity and involvement before the birth of the baby. Illustrating the intervention process, the current study presents two case studies of expectant fathers receiving VIPP‐PRE (clinical trial registration NL62696.058.17). The VIPP‐PRE program is described along with the individual dyads’ prenatal video fragments and feedback specific for each father‐fetus dyad. In addition, changes in paternal sensitivity and involvement levels are presented, as well as fathers’ and intervener's evaluation of the intervention. VIPP‐PRE promises to be a feasible short‐term and potentially effective parenting intervention for expectant fathers. Currently, a randomized controlled trial (RCT) is under review that systematically investigates the efficacy of the VIPP‐PRE. Here we aim to provide further information on the intervention process, as well as fathers’ and intervener's evaluations of this process, and the benefits of using ultrasound imaging in a parenting intervention.

## Resumen

Aunque las intervenciones de crianza incluyendo a los papás en espera son escasas, las mismas producen resultados prometedores. La Intervención Prenatal de Información en Video para promover la Crianza Positiva (VIPP‐PRE) es una intervención recientemente desarrollada, la cual es manejada a mano y personalizada, con el objetivo de mejorar la sensibilidad y la participación paterna antes del nacimiento del bebé. Demostrando el proceso de intervención, el presente estudio presenta dos casos de estudio de papás en espera que reciben VIPP‐PRE (registro de ensayo clínico NL62696.058.17). El programa VIPP‐PRE se describe junto con los fragmentos e información del video prenatal de las díadas individuales específicas para cada díada papá‐feto. Adicionalmente, se presentan los cambios en los niveles de sensibilidad y participación paterna, así como también la evaluación de la intervención por parte de los papás y de quien condujo la intervención. VIPP‐PRE promete ser una intervención de crianza factible a corto plazo y potencialmente efectiva para papás en espera. Actualmente, se encuentra bajo revisión un ensayo controlado al azar (RCT) que sistemáticamente investiga la efectividad de VIPP‐PRE. Aquí nos proponemos ofrecer mayor información sobre el proceso de intervención, así como también las evaluaciones que del proceso hicieron los papás y quien condujo la intervención, y los beneficios de usar las imágenes de ultrasonido en una intervención de crianza.

## Résumé

Bien que les interventions de parentage in incluent des futurs pères soient rares, elles donnent des résultats prometteurs. L'Intervention de Retour‐Vidéo Prénatale pour promouvoir le Parentage Positif (abrégé en anglais VIPP‐PRE) est une intervention récemment développée. Elle est à la fois manualisée et personnalisée, se donnant pour but de renforcer la sensibilité et l'engagement avant la naissance du bébé. Illustrant le processus d'intervention cette étude présente deux études de cas de futurs pères recevant la CVIP‐PRE (enregistrement des essais cliniques NL62696.058.17). Le programme VIPP‐PRE est décrit ainsi que les fragments vidéo prénatale des dyades individuelles et le retour spécifique pour chaque dyade père‐fœtus. De plus les changements dans la sensibilité paternelle et dans les niveaux d'engagement sont présentés, ainsi que l’évaluation des pères et des intervenants de l'intervention. La VIPP‐PRE promet d’être une intervention de parentage viable à court terme et potentiellement efficace pour les futurs pères. Pour l'instant un essai contrôlé randomisé est à l’étude, étudiant systématiquement l'efficacité de la VIPP‐PRE. Ici nous nous donnons pour but d'offrir des informations plus amples sur le processus d'intervention, ainsi que sur les évaluations du processus faites par les pères et les intervenants, et sur les bénéfices qu'il y a à utiliser les images ultrasons dans une intervention de parentage.

## Zusammenfassung

Die pränatale Video‐Feedback‐Intervention zur Förderung positiver Elternschaft für werdende Väter (VIPP‐PRE): Zwei Fallstudien

Es nur wenige Elterninterventionen, die für werdende Väter konzipiert sind, doch die Ergebnisse solcher Interventionen sind vielversprechend. Die pränatale Video‐Feedback‐Intervention zur Förderung positiver Elternschaft (Prenatal Video‐feedback Intervention to promote Positive Parenting; VIPP‐PRE) ist eine kürzlich entwickelte Intervention, die sowohl manualisiert als auch personalisiert ist und darauf abzielt, die väterliche Sensibilität und Beteiligung vor der Geburt des Kindes zu verbessern. Zur Veranschaulichung des Interventionsprozesses werden in der vorliegenden Arbeit zwei Fallstudien von werdenden Vätern vorgestellt, die VIPP‐PRE durchlaufen haben (clinical trial registration NL62696.058.17). Das VIPP‐PRE‐Programm wird zusammen mit den pränatalen Videofragmenten der einzelnen Dyaden und dem für jede Vater‐Fötus‐Dyade spezifischen Feedback beschrieben. Darüber hinaus werden Veränderungen in der väterlichen Sensibilität und dem Grad der Beteiligung sowie eine Interventionsevaluation durch die Väter und die Durchführenden dargestellt. VIPP‐PRE verspricht eine machbare, kurzfristige und potenziell wirksame Elternintervention für werdende Väter zu sein. Derzeit befindet sich eine randomisierte kontrollierte Studie (RCT) im Review, die systematisch die Wirksamkeit von VIPP‐PRE untersucht. In der vorliegenden Arbeit möchten wir weitere Informationen über den Interventionsprozess, die Bewertung dieses Prozesses durch die Väter und die Durchführenden sowie über die Vorteile der Verwendung von Ultraschallbildern in einer Elternintervention geben.

## 抄録

未来の父親に肯定的な子育てを促進するための出生前ビデオフィードバック介入 (VIPP‐PRE) の2事例

未来の父親向けの育児介入は、非常に少ないが、有望な結果が得られている。肯定的な子育てを促進するための出生前ビデオフィードバック介入 (VIPP‐PRE) は、子どもが生まれる前に父親の感受性と関与を高めることを目的とした、マニュアル化も個別化もされ、最近開発された介入法である。本研究では、VIPP‐PRE (臨床試験登録NL62696.058.17) を受けた未来の父親の2つの事例を紹介し、介入の過程を説明する。VIPP‐PREプログラムについて、各二人組の出生前のいくつかの短いビデオと各父親‐胎児の組への個別フィードバックとともに説明する。さらに、父親の感受性と関与レベルの変化、および父親と介入者の介入に対する評価も提示した。VIPP‐PREは、未来の父親にとって短期的に実現可能なかつ効果的な育児介入となる可能性があることが期待される。現在、VIPP‐PREの有効性を系統的に検討する無作為化比較試験 (RCT) が検討されている。ここでは、介入のプロセス、それに対する父親と介入者の評価、子育て介入に超音波画像を使用することの利点について、さらなる情報を提供することを目的とする。

## 摘要

尽管包括准爸爸在内的育儿干预措施很少, 但它们产生了可喜的成果。促进积极育儿的“产前视频反馈干预” (VIPP‐PRE) 是最近开发的的一项干预措施, 它既程式化又个性化, 旨在提高婴儿出生前父亲的敏感性和参与度。为了说明干预过程, 本研究介绍了两个接受VIPP‐PRE (临床试验注册NL62696.058.17) 的准爸爸的案例研究。描述了VIPP‐PRE程序与各个二人组的产前视频片段, 以及针对每个父亲‐胎儿二人组的反馈。此外, 还介绍了父亲敏感性和参与度的变化, 以及父亲和干预者对干预措施的评价。VIPP‐PRE有望成为一种短期可行的、潜在有效的准爸爸育儿干预措施。目前, 一项系统研究VIPP‐PRE有效性的随机对照试验 (RCT) 正在审查中。在这项研究中, 我们旨在提供有关干预过程的更多信息, 父亲和干预者对这一过程的评价, 以及在育儿干预中使用超声成像的好处。

## ملخص

تدخل التغذية الراجعة بالفيديو قبل الولادة لتعزيز التربية الإيجابية للآباء المنتظرين (PRE‐VIPP) دراستان حالة

على الرغم من ندرة تدخلات التربية بما في ذلك الآباء المنتظرون ، إلا أنها تسفر عن نتائج واعدة. يعد تدخل التغذية الراجعة بالفيديو قبل الولادة لتعزيز التربية الإيجابيةPRE) ‐(VIPP تدخلاً تم تطويره مؤخرًا ، يدويًا وشخصيًا على حد سواء ، بهدف تعزيز حساسية الأب ومشاركته قبل ولادة الطفل. لتوضيح عملية التدخل ، تقدم الدراسة الحالية دراستي حالة للآباء المتوقعين الذين يتلقون (PRE‐VIPP) (تسجيل التجربة السريرية NL62696.058.17). يتم وصف برنامجPRE ‐VIPP مع مقاطع فيديو ما قبل الولادة الخاصة بالثنائيات والتعليقات الخاصة بكل ثنائي من الأب والجنين. بالإضافة إلى ذلك ، يتم عرض التغييرات في حساسية الأب ومستويات المشاركة ، وكذلك تقييم الآباء والقائمين على البرنامج للتدخل. يعد برنامجPRE ‐VIPP بأن يكون تدخلاً عمليًا قصير الأجل وفعالاً للآباء المنتظرين. حاليًا ، تجري مراجعة تجربة معشاة ذات شواهد(RCT) تحقق بشكل منهجي في فعالية هذا البرنامج. نهدف في هذه الورقة إلى تقديم مزيد من المعلومات حول عملية التدخل ، بالإضافة إلى تقييمات الآباء والمتدخلين لهذه العملية ، وفوائد استخدام التصوير بالموجات فوق الصوتية في التدخلات الخاصة بالتربية الأبوية.

## 1 INTRODUCTION

Parenting sensitivity is defined as the awareness and accurate interpretation of infant signals, accompanied by a prompt and adequate response to such signals (Ainsworth et al., 1974). Correlational and experimental studies found parenting sensitivity to be of crucial importance for infant attachment security, that is, the development of a “secure base relationship” in which the infant uses the parent as a secure base from which to explore his environment and a safe haven to turn to in times of distress (Bakermans‐Kranenburg et al., 2003; De Wolff & Van IJzendoorn, 1997; Lucassen et al., 2011). The mother‐child dyad has long been the main focus of attachment related studies, and only few early studies took other attachment relationships explicitly into account (e.g., Van IJzendoorn et al., 1992). The predominant thought in the recent literature is however that children can have multiple important attachment relationships, with father being the most evident besides mothers (Dagan & Sagi‐Schwartz, 2018). Paternal sensitivity is known to contribute substantially and independently from maternal sensitivity to children's cognitive development (Tamis‐LeMonda et al., 2004; Towe‐Goodman et al., 2014). Moreover, fathers provide unique learning opportunities for their children by combining their characteristic “arousing” play with sensitive parenting behavior (Hazen et al., 2010; Paquette, 2004).

**KEY FINDINGS**

- Two case studies revealed mixed results in the enhancement of paternal sensitivity and involvement with the Video‐feedback Intervention to promote Positive Parenting‐Prenatal (VIPP‐PRE)
- In general, the two fathers positively evaluated VIPP‐PRE and reported more insight in their communication and relationship with the baby
- The use of ultrasound imaging in parenting interventions may open new ways to support parents in the earliest stages of parenthood

**STATEMENT OF RELEVANCE**

Fathers’ parenting is relevant for child mental health and development. The prenatal period seems to offer great opportunities for the early enhancement of paternal parenting. Yet, few parenting interventions focus on the prenatal period or include fathers. VIPP‐PRE promises to be a feasible short‐term and potentially affective parenting intervention for expectant fathers. The use of live ultrasound images may have important benefits for fathers and children.

In addition to sensitivity, fathers’ involvement in play and caregiving was found to be positively related with infant‐father attachment security (Brown et al., 2018) and to contribute significantly to a variety of positive child outcomes, such as social competence and cognitive development (Dubowitz et al., 2001). Paternal sensitivity and involvement both appear to have its origin in the prenatal phase. For example, fathers’ prenatal sensitive caretaking of an infant simulator was found to predict paternal sensitivity towards their infant 6 weeks post birth (Hechler et al., 2019), indicating that sensitive caregiving behaviors during pregnancy can be measured and have predictive power for the postnatal phase. Other studies demonstrated that fathers who were more involved during pregnancy, were more engaged in infant care after birth (Cabrera et al., 2008; Zvara et al., 2013). Thus, prenatal involvement of fathers is predictive for the level of engagement when the child is born. Bearing this in mind, the prenatal period seems to offer opportunities for the early enhancement of paternal parenting.

At the same time, perinatal care remains primarily focused on mothers (Lever Taylor et al., 2018) and few parenting interventions include fathers. Moreover, existing interventions mainly address the postnatal phase, whereas prenatal interventions may be especially beneficial for fathers. Paternal participation in prenatal education programs has been suggested to be beneficial for later father involvement and – possibly – child well‐being (Bronte‐Tinkew et al., 2007), and expectant fathers have expressed a need for support and guidance, preparing them for parenthood (Palsson et al., 2017). In addition, fathers report barriers when seeking to be engaged in their partner's pregnancy (Steen et al., 2012; Widarsson et al., 2015) and they have described feeling distant and having a harder time bonding to their unborn baby due to the fact that they do not directly experience its physical presence (Van Bakel et al., 2013; Vreeswijk et al., 2014).

Although the fetal period is understudied in psychological research, evidence suggests that fetuses actively process sensory stimuli (Reid & Dunn, 2021). For example, fetal movements in response to sounds coming from outside the abdomen (as visualized by ultrasound imaging) were found from 19 weeks of pregnancy onwards, demonstrating that fetuses are able to hear at that gestational age (Hepper & Shahidullah, 1994). Also, fetal heart rate was found to increase when hearing mother's voice, as well as father's voice after daily exposure for a week prior to testing, indicating that fetuses respond to familiar voices (Lee & Kisilevsky, 2014). Moreover, fetuses were found to touch the uterus wall longer in response to mothers’ touch on her abdomen, and this increased from the second to third trimester of pregnancy (Marx & Nagy, 2017). This could be valuable information for expectant fathers, potentially encouraging them to engage in the pregnancy and develop a personal relationship with the unborn infant.

To our knowledge, only two parenting interventions exist that include fathers in the prenatal phase. One of these interventions is “Growing as a Couple and Family,” described by Bryan (2000). In three prenatal classes, this intervention aims to enhance expectant parents’ mutual knowledge and understanding of newborn babies, their competence and confidence in their new role as a parent, and the quality of parent‐infant interactions after birth. Expectant parents are shown videos of unknown parent‐infant interactions, followed by discussions on co‐parenting, enjoyment, and mutual support. Furthermore, parents receive information on infant interactive abilities, and on growth and maturity patterns in the first three months after birth (e.g., growth spurts, sleep rhythms). The intervention has been shown to positively affect couples’ response to child distress. For fathers, it also increased affective support in interaction with their infant (e.g., smiling, praising, and excitement over the child's efforts).

The other prenatal intervention that includes fathers is “Parenting Together,” described by Doherty et al. (2006). This group intervention for couples encompasses eight educational sessions starting in the second trimester of pregnancy and continuing till five months after birth. An individual home visit is followed by seven group sessions in a clinic. By means of mini‐lectures, discussions, and standardized videotapes, the intervention aims to improve father–child interaction quality and to increase paternal involvement in their children's lives. The intervention was found effective in improving fathers’ interaction with their infants (i.e., more warmth and emotional support, less intrusiveness, more positive affect, and more dyadic synchrony) and the level of paternal involvement during workdays (i.e., the time father spent in proximity to the child although he was not necessarily interacting with the child).

These two prenatal parenting interventions that include fathers yielded promising results. However, themes like father‐fetal bonding, pregnancy engagement, and emotionally attuned interaction between father and the unborn baby seem to be neglected. This is unfortunate given the difficulties that fathers can experience in bonding to the fetus (Van Bakel et al., 2013; Vreeswijk et al., 2014) and the finding that fathers’ prenatal involvement and sensitivity predict paternal parenting quality in the postnatal phase (Cabrera et al., 2008; Hechler et al., 2019; Zvara et al., 2013), which in turn has been associated with a variety of positive child outcomes (Brown et al., 2018; Dubowitz et al., 2001; Hazen et al., 2010; Tamis‐LeMonda et al., 2004). Furthermore, group discussions tend to be of a more general nature and the interventions so far make use of standardized videos of unknown parent‐infant dyads. As such, these interventions possibly fail to address unique and personal father–baby contact. Bakermans‐Kranenburg et al. (2003) found that interventions using personalized video feedback were more effective in improving parenting sensitivity when compared to interventions that did not use this method. Video feedback enables parents to reflect on their children's perspectives and on their own parenting behaviors (Juffer et al., 2017), which might be essential for the enhancement of sensitivity.

The scarcity of prenatal parenting programs for fathers, whilst this period holds important implications for postnatal caregiving quality, suggests that there is a need for new evidence‐based interventions. Hence, a short‐term prenatal intervention for fathers was developed: the Prenatal Video‐feedback Intervention to promote Positive Parenting (VIPP‐PRE). VIPP‐PRE aims to improve expectant fathers’ postnatal sensitivity to their baby's needs and enhance paternal involvement by using live ultrasound images as an aid to visualize father‐fetus interactions. As an important feature and complementary to existing interventions, VIPP‐PRE addresses the unique relation between father and fetus by using videos of each dyad, as well as personalized feedback, while focusing on parental sensitivity and pregnancy involvement.

VIPP‐PRE follows a protocol adapted from the original Video‐feedback Intervention to promote Positive Parenting (VIPP) program (Juffer et al., 2008); and is founded on principles of attachment theory (Ainsworth et al., 1974; Bowlby, 1997). In a series of randomized controlled studies, the VIPP and Video‐feedback Intervention to promote Positive Parenting and Sensitive Discipline (VIPP‐SD) programs have been found effective in improving parenting sensitivity in mothers and professional caregivers (Juffer et al., 2017), starting in the first year of life (Klein Velderman et al., 2006). Furthermore, the program has successfully been adapted for non‐clinical and clinical populations (Barone et al., 2018; Iles et al., 2017; Poslawsky et al., 2015; Van Zeijl et al., 2006). A meta‐analytic review including 12 randomized‐controlled trials (RCTs) found a combined effect size of *d *= .47 for enhanced sensitivity (Juffer et al., 2017). Although the majority of studies focused on mothers, a pilot study in fathers yielded promising results, with fathers reporting that participation in the VIPP had a substantial impact on their understanding of, communication with, and relationship with their baby (Lawrence et al., 2013).

Previously, the authors have demonstrated the feasibility of the VIPP‐PRE intervention, as well as positive evaluations of the intervention from both fathers and ultrasound technicians delivering the intervention (Alyousefi‐van Dijk et al., 2021). In this case study, we elaborate on the VIPP‐PRE intervention process by presenting two case studies of expectant fathers receiving VIPP‐PRE to support interaction with their unborn baby. The VIPP‐PRE program is described along with the individual dyads’ prenatal video fragments and feedback specific for each father‐fetus dyad. In addition, paternal sensitivity and involvement levels are discussed, as well as fathers’ and intervener's evaluation of the intervention. In doing so, the current study aims to provide further information on the intervention process and how ultrasound imaging can be used in a prenatal parenting intervention.

## 2 MATERIALS AND METHODS

### 2.1 Procedure and measures

As part of a study on the transition to fatherhood (clinical trial registration NL62696.058.17), first‐time expectant fathers were randomly assigned to either an experimental group receiving VIPP‐PRE, or a control condition consisting of three telephone calls during which the development of the fetus, pregnancy progress, and upcoming fatherhood were discussed. Participants in the VIPP‐PRE group and their partners came to a prenatal health clinic (Verloskundig Centrum de Poort in Leiden, the Netherlands) three times during pregnancy, preferably between 20 and 30 weeks of gestation. To assess the effects of VIPP‐PRE on paternal sensitivity and involvement, participants took part in three research visits: one baseline lab visit (prenatal), one lab visit after completion of the intervention or control condition (prenatal), and one home visit approximately 9 weeks after birth (postnatal). The study was approved by the Ethics Committees of the Leiden University Medical Centre (NL62696.058.17, P17.216) and of the Department of Education and Child studies at Leiden University (ECPW2017/170), and was carried out in accordance with The Code of Ethics of the World Medical Association (Declaration of Helsinki). All participants and their partners gave informed consent. Participants received financial compensation for each lab or home visit (up to €100 in total) plus travel allowance, and an extra €10 after the completion of the study if they completed at least 80% of the questionnaires that were sent to participants following each research visit.

During the intervention process, a log was kept by the intervener in order to evaluate the progress and describe any particularities regarding the sessions (e.g., visibility of the baby, fathers’ reaction to the session). At all research visits, participants were observed while taking care of an infant simulator (prenatal visits) or playing with their own baby (postnatal visit) for ten minutes. Parenting sensitivity was assessed by using the Ainsworth Sensitivity Scale (Ainsworth et al., 1974) with scores ranging from 1 to 9 and higher scores reflecting more sensitivity. In the week following each research visit, a smartphone application measured fathers’ involvement with and accessibility to the baby (e.g., “In the past 15 min, have you interacted with your baby?”). Scores (0 = no, 1 = yes) over 42 measure moments were averaged, yielding a score that ranged from 0 to 1 with higher scores indicating more involvement and more time spent in proximity to the baby. VIPP‐PRE sessions were evaluated by an online questionnaire completed by participants at home following the second research visit. Fathers were asked whether they thought the intervention gave them more insight in their (1) relationship with the baby, (2) understanding of the baby, (3) communication with the baby, and (4) the baby's feelings. Items were rated on a 5‐point Likert scale (1 = *not at all*, 2 = *a little*, 3 = *relatively much*, 4 = *much*, 5 = *very much*) with higher scores reflecting increased insight into the unborn baby. On the same 5‐point Likert scale, the helpfulness of the intervention was assessed. One question evaluated fathers’ perceived interaction with the intervener, rated on a Likert scale ranging from 1 (*very unpleasant*) to 5 (*very pleasant*). In two open‐ended questions, fathers reported what they liked most and least about the intervention sessions.

### 2.2 Case selection

Expectant fathers were included in the study in the case of first‐time uncomplicated singleton pregnancies. Moreover, healthy development of the fetus had to be confirmed by a standardized medical ultrasound at 20 weeks of gestation in regular health care services. Exclusion criteria were genetic abnormalities in the participant's or partner's family, partner's dependency on alcohol, tobacco, or illicit drugs during pregnancy, or alcohol or drugs abuse of the expectant father. For the current study, eligible cases were participants that were randomized to the intervention condition, with the first author being the intervener, and with complete intervention sessions. A total of 73 expectant fathers participated in the study, of whom 39 were randomized in the intervention condition. Eighteen of these interventions were conducted by the first author as intervener and half of those were missing any pertinent prenatal or postnatal data. As VIPP‐PRE aims to enhance parenting sensitivity, the two cases were then selected on the basis of their baseline sensitivity scores with an infant simulator as observed during the first lab visit. We selected one father with an average score on sensitivity, representing the current study's sample, and one father with a below average score on sensitivity, representing a group of fathers for whom the intervention in future applications of VIPP‐PRE might be particularly important.

### 2.3 The VIPP‐PRE program

VIPP‐PRE consists of three sessions with approximately 1–2 weeks in between sessions. The first session lasts half an hour, and the second and third sessions last approximately 1 hour. Each individual intervention process follows the same structure and addresses specific themes (see Table 1). The first two sessions focus on building a working relationship with the expectant father and emphasize positive father‐baby interactions. The third session focuses on actively improving parenting behavior by means of “corrective messages” (i.e., carefully discussing insensitive behavior and suggesting a more sensitive alternative). Upon request of the ethics committee, all sessions in this study took place in a prenatal health clinic. Here, an ultrasound technician provided live and recognizable images of the baby.

**TABLE 1:** Themes in the VIPP‐PRE program

**Session**	**Themes live feedback**	**Themes video‐feedback**
**1**	Exploration and attachment (i.e., exploratory and attachment related behaviors of the baby are discussed, as well as parents’ sensitive responding towards these behaviors)	
**2**	Speaking for the baby (i.e., verbalizing recognizable behavior of the baby and carefully interpreting its emotions or sensations)	Exploration and attachment
**3**	Sensitivity chains (i.e., a baby's signal, followed by an appropriate response of the parent and a positive reaction of the baby)	Speaking for the baby and sensitivity chains

#### 2.3.1 Introduction

In the first session, expectant parents are informed about the intervention process. They are told that recordings will be made of father interacting with his baby and that, for this purpose, father will be asked to do various things such as reading and singing. It is explained that, although this can be somewhat difficult, they should act as they would normally do. Parents are informed that the recordings will be reviewed with the father during the next session, and that the intervener will then provide feedback based on her experiences with and knowledge of children. The intervener also discusses that father is an expert on *his* baby, as he knows the baby best. In addition, parents are informed that the ultrasound images will not serve as a prenatal screening or to answer questions regarding the fetus’ health or pregnancy abnormalities. Finally, it is explained that during the recordings, mother will be asked to read a magazine and the intervener will solely address father, for the purpose of facilitating a one‐to‐one moment for father and baby as much as possible. Before starting with the intervention activities, ultrasound images are viewed with both parents in order to meet mothers’ wishes to see their unborn babies as well.

#### 2.3.2 Filming

During the first part of each session, the intervener records brief videos of father–baby interactions using a mobile ultrasound system (Philips Lumify 2017, Best, The Netherlands). Recordings include the ultrasound images of the unborn baby, and a frontal view of father, seated beside the mother's abdomen. In the first session, two fragments are recorded. First, fathers are asked to read for about three minutes from a children's book brought by the intervener. Second, fathers are invited to interact for about two minutes with their unborn child by gently touching their partner's abdomen. During the second session, fathers are asked to sing for and speak to their baby for 2 and 3 min, respectively. When fathers struggle to come up with a topic, the intervener suggests telling the bay what they will be doing together after birth. In the third session, fathers are asked to interact in free play with their infants for 5 min.

#### 2.3.3 Live feedback

While recording, the intervener provides live feedback by using “speaking for the baby” techniques, that is, verbalizing recognizable behavior of the baby and carefully interpreting its emotions or sensations (*“Your baby is yawning, maybe he/she is tired”*). In addition, father is stimulated to “read” his baby's signals and behave accordingly. At all times, the intervener does not interfere with ongoing interactions. Father also receives information on fetal development corresponding to the gestational age, and sensory abilities of his unborn baby. In the first session, it is explained to the father that, at a certain stage, babies are capable of hearing voices coming from outside the abdomen and can recognize father's voice (Lee & Kisilevsky, 2014). In the second session, father is told that babies can remember rhythms and music during pregnancy and even after birth when heard regularly (Granier‐Deferre et al., 2011). In the third session, the intervener explains that – as pregnancy progresses – babies are more able to feel and respond to touch as the mothers’ abdomen skin gets thinner, the level of amniotic fluid decreases and the baby's nervous system develops (Marx & Nagy, 2017).

When applicable, information is provided on the session's corresponding theme (see Table 1). In the first session, it is explained to father that his baby is often either exploring (e.g., playing with its toes) or seeking contact with its caregiver (i.e., attachment related behaviors). The importance of parents providing learning opportunities, as well as being a safe haven for their child, is emphasized. The second session's theme (“speaking for the baby”) is integrated in the video feedback and not explicitly discussed. In the third session, “sensitivity chains” (i.e., a baby's signal, followed by an appropriate response of the parent and a positive reaction of the baby) are discussed. The intervener explains that adequate responses to the baby's signals make the baby feel understood and help him/her build trust in self and in the parent.

#### 2.3.4 Script‐based feedback

In the second and third sessions, the videos that were recorded during the previous session are shown to father. The intervener presents the full recordings, while pausing at relevant moments. Using a prepared “script”, the intervener then comments on these fragments (e.g., “speaking for the baby,” reinforcing sensitive and emotionally attuned behavior of the father, emphasizing positive interactions). Comments are based on the themes and guidelines in the VIPP‐PRE‐manual, but also personalized, addressing each specific father‐baby dyad and unique fragment. Since visualization and interpretation of the baby's behavior is limited, the intervener is careful in interpreting the images (e.g., *“Your baby stopped moving. Maybe he/she is listening to your voice right now. At this stage, they are certainly capable of hearing voices coming from outside the mother's abdomen.”*). Although interpretation of fetal responses as attachment related behavior may seem presumptive, it provides a window to discuss the distinction between children's proximity‐seeking behavior on the hand, and exploratory behavior on the other hand, and how to respond sensitively to each of these infant signals, now as well as after the child is born. During the discussion of the recordings, the intervener offers father opportunities to actively contribute by inviting him to comment on the recordings and his baby's behavior. Furthermore, empathy is shown for both father (*“I can imagine it is still difficult for you to see what your baby is doing as he/she is not born yet”*) and baby (*“It takes a lot of energy growing so much every day”*). Finally, the intervener stresses the importance of spending time interacting with the baby, and father is invited to regularly make contact with his baby outside of the appointments.

#### 2.3.5 Intervener

The intervener (i.e., first author) was a behavioral scientist trained in the Video‐feedback Intervention to promote Positive Parenting and Sensitive Discipline (VIPP‐SD). Subsequently, the intervener was trained in the VIPP‐PRE intervention by the second and last authors of this paper. The intervener completed both intervention cases described in this study.

## 3 CASE 1

### 3.1 Participant description

Daniel (name and details are changed for anonymity) is 30 years old, was born in the Netherlands, holds a bachelor's degree, and is living together with his partner who was pregnant with their first child, a son. There are no known genetic abnormalities in the families of both expectant parents, and a healthy physical development of the unborn baby was established on a standard 20‐weeks ultrasound scan in a regular health care setting. Intervention sessions took place at 27 weeks and three days, 28 weeks and three days, and 34 weeks and 1 day of gestation.

### 3.2 Course of treatment

#### 3.2.1 Session 1

*Live feedback: Exploration and attachment*. During the first session, Daniel was informed that in week 27 of the pregnancy, his baby will open his eyes for the first time and his cranial nerves are specializing into different senses (e.g., hearing, taste). While recording, the intervener provided subtitles for the baby's behavior by mentioning the baby moving his arms and touching his mouth with his hands, and identified these fragments as exploratory behavior. Moments when the baby was calm were interpreted as attachment related behaviors. The intervener then carefully commented on the baby's intentions, suggesting he was resting as he had gotten tired of moving or, alternatively, that he was attentively listening to his father's voice.

*Profile*. Based on the recordings from the first session, the intervener made a profile of the father–baby interaction. The intervener had noted that Daniel used a warm and gentle voice when speaking to his baby and showed an appropriate pace in speech and actions. Also, two profile items were selected from the list in the VIPP‐PRE manual, consisting of nine profile points that describe behavior of a sensitive parent (see Appendix A). These profile items specify opportunities for change in each unique parent‐fetus dyad, and are used as a guideline for the personalized feedback. For Daniel, the first profile item that was selected was “understanding of what moves the baby and showing empathy,” as the intervener deemed this to be difficult for Daniel. The second item was “noticing and correctly interpreting the behavior of the baby, and reacting correctly to it,” as this was considered to be an area in which Daniel could develop.

#### 3.2.2 Session 2

*Live feedback: Speaking for the baby*. Daniel was told that in week 28 of the pregnancy, his baby is able to blink, he is coughing regularly, and his sight is rapidly developing, as well as the billions of cells in his brain. During the recordings, the intervener used “speaking for the baby” techniques by verbalizing the baby being very calm and suggesting he was napping or enjoying the pleasant voice of his father. Also, the baby's sleep‐wake rhythm was discussed.

*Script‐based feedback: Exploration and attachment*. To give Daniel more insight into and understanding of his baby's behavior, fragments of exploration and attachment were shown and verbalized by the intervener. The baby touching his mouth with his hand, moving his legs, sucking on his hand, and moving his head up and down, were described as exploratory behaviors. It was explained that his baby was probably not able to hear his voice at those moments, as babies of that gestational age are not yet capable of doing multiple things at the same time. Additional information on child development was shared: babies are very curious and need to discover their surroundings in order to learn and get to know the world around them. Fragments showing a calm baby were carefully interpreted as attachment related behaviors. The intervener explained that hearing Daniels familiar voice encompasses security and comfort, because as a father, he is a safe haven for his baby. Emotionally attuned behaviors were emphasized by presenting a fragment of Daniel touching his partner's abdomen in a gentle and appropriate manner, in order not to disturb or wake his baby. The intervener complimented Daniel and explained the importance of adequately responding to infant cues. In addition, Daniel was encouraged to comment on his baby's behavior (e.g., *“What do you think he is doing here?”*) and future interactions between Daniel and his baby were encouraged.

#### 3.2.3 Session 3

*Live feedback: Sensitivity chains*. Daniel was told that in week 34 of the pregnancy, his baby has less space to move around, and that nails are growing on his fingertips. During the recordings, the intervener commented on the baby drinking amniotic fluid and explained that, in this stage of the pregnancy, babies are able to taste different flavors. Daniel was asked whether he could feel his baby moving around, and if he had ever seen his baby in this position, with his hand in front of his face. Finally, it was discussed that sometimes babies do respond to touch whereas at other moments they do not, as they are too busy exploring their environment.

*Script‐based feedback: Speaking for the baby and sensitivity chains*. Behavior of the baby (e.g., the baby holding his ear and moving his hands) was verbalized by the intervener. It was explained that babies need time to learn and that it is important for parents to adjust their pace to their baby's pace. The intervener invited father to comment on his baby's behavior (*“Do you think your baby woke up or moved in his sleep?”*). Related to fragments of the baby's behavior (e.g., moving or playing with his hands) or father's response (e.g., talking to the baby), information on “sensitivity chains” was provided. Also, the intervener explained that some situations (e.g., play) provide excellent opportunities for parents to follow the baby's lead and emphasized the importance of parents verbalizing the baby's feelings, wishes, and needs.

### 3.3 Evaluation

#### 3.3.1 Intervener's experience

The intervener described Daniel as enthusiastic, cooperative, and involved. He clearly enjoyed seeing his baby on the ultrasound images. The first session stimulated Daniel to make contact with his baby on a more regular basis, as he started to read to his baby every evening following the session. With regard to the first profile item (understanding of what moves the baby and showing empathy), behavior that indicated Daniel trying to empathize with his baby was highlighted by the intervener. Such behaviors, for example father's comments on his baby's behavior and sensations (e.g., *“Enjoying a good massage”*) and speaking on behalf of the baby (e.g., *“This is the moment, now I can play”*), appeared to occur increasingly throughout the intervention. Also with regard to the second profile issue (noticing and correctly interpreting the behavior of the baby, and reacting correctly to it), the intervener emphasized moments of sensitive parenting behavior. For example, a fragment where Daniel verbalized his baby's perspective (“*I don't want to tease you because you seem so comfortable”*) and gently touched his partner's abdomen, indicating he observed and interpreted his baby's behavior and acted upon this. The intervener felt that Daniel showed great interest in and was open to the feedback that was provided at such moments.

#### 3.3.2 Father's evaluation

Daniel indicated that he gained much more insight in the relationship with the baby and more insight in his communication with the baby, with scores of 4 and 3 (out of 5), respectively. Furthermore, he reported much more understanding of the baby and a little more understanding of his baby's feelings, with scores of 4 and 2, respectively. Daniel considered the intervention sessions to be helpful (score 4) and rated the interaction with the intervener as very pleasant (score 5). What Daniel liked most about the sessions was “*having a moment to admire my son. To see that he responded to my presence and to follow his development better than without the sessions. These were precious moments to me*.*”* What Daniel liked least about the sessions was the fact that he was not able to share his experience with his partner as much as he had wanted to.

### 3.4 Sensitivity and involvement assessments

Daniel's parenting sensitivity scores ranged from 5 at baseline, to 5.5 after completion of the intervention and 9 post birth. These scores demonstrated a slight increase in sensitivity after completion of VIPP‐PRE, and a large increase after birth. In addition, Daniel scored .65 at baseline, .63 after completion of the intervention, and .87 post birth for involvement with the baby, and .54, .74, and .91 for accessibility, respectively. These scores, derived from a self‐rated smartphone application, indicate the number of times that Daniel was thinking about or interacting with his child (involvement), or was in proximity to his child (accessibility), relative to the total number of times that he was asked if he was. For both, this entailed an increase from baseline to post birth. For accessibility specifically, an increase was also visible immediately after completion of the intervention. Of course, it should be noted that these scores concern one case study and are not compared to a control condition. Therefore, caution is required when interpreting these results.

## 4 CASE 2

### 4.1 Participant description

Liam (name and details are changed for anonymity) is a 34‐year‐old expectant father, cohabiting with his partner who was pregnant with their first child, a daughter. Liam was born in the Netherlands and holds a bachelor's degree. There are no known genetic abnormalities in families of both expectant parents, and a healthy psychical development of the fetus was established on a standard 20‐weeks ultrasound scan in regular health care. Intervention sessions took place at 29 weeks and 1 day, 30 weeks and 6 days, and 32 weeks and 2 days of gestation.

### 4.2 Course of treatment

#### 4.2.1 Session 1

*Live feedback: Exploration and attachment*. During the first session, Liam was told that, in week 29 of the pregnancy, his baby can distinguish sweet and sour flavors in the amniotic fluid, and her eyelashes and eyebrows are now fully developed. During the recordings, the intervener commented on the baby's behavior (e.g., moving her mouth, being calm), and suggested the baby felt comfortable. Liam was asked whether he could feel the baby's movements, and information was provided on exploratory and attachment related behaviors, as well as his baby's ability to respond to father.

*Profile*. The intervener had noted that Liam was engaged in the session, yet seemed to be feeling a bit awkward performing the tasks. Liam was interested in the ultrasound images of his baby, but at times seemed to miss the information that was provided by the intervener. The two profile items that were selected as opportunities for change were “understanding of what moves the baby and showing empathy,” and “noticing of signals and observing the baby” as these were considered to be first steps for Liam in developing sensitivity toward his baby's signals.

#### 4.2.2 Session 2

*Live feedback: Speaking for the baby*. Liam was told that in week 30 of the pregnancy, his baby is able to grab the umbilical cord and touch her own face, can get the hiccups, and is able to dream, as well as separate light from darkness. During the recordings, the intervener used “speaking for the baby” techniques by mentioning the baby being calm, and suggesting she was relaxing and enjoying her father's voice. In addition, sensations of babies in the uterus were discussed (e.g., perfect temperature, continuous food supply).

*Script‐based feedback: Exploration and attachment*. Fragments of exploratory behavior were shown and verbalized by the intervener: the baby sucking on her hand, pushing her hand against the uterus wall, moving her feet, and turning her head. Fragments of the baby becoming calm after hearing Liam's voice, and turning her head towards father's touch, were interpreted as attachment related behaviors. The intervener carefully suggested that Liam's baby was responding to her father's voice and touch, and it was explained that father is a safe haven for his baby, providing the baby security and comfort when being in proximity. In addition, emotionally attuned behavior of Liam was emphasized by showing a fragment in which Liam gave his baby time to explore her surroundings, and did not interfere. Also, the intervener complimented Liam on verbalizing his actions for his baby, as this will support the baby in learning and getting familiar with the world around her. In order to actively involve father in the intervention process, several questions were asked, e.g., *“Have you ever noticed her becoming calm when you started talking?”*. Finally, contact between Liam and his baby were stimulated by emphasizing how Liam's baby enjoyed her father's attention.

#### 4.2.3 Session 3

*Live feedback: Sensitivity chains*. Liam was told that, in week 32 of the pregnancy, moving around keeps getting more challenging for his baby, as she keeps on growing. During the recordings, the intervener verbalized Liam's baby moving around, and – at a later stage – being calm. She invited Liam to comment on his baby's behavior by asking him whether he thought she fell asleep or stopped moving for another reason.

*Script‐based feedback: Speaking for the baby and sensitivity chains*. Fragments of the baby taking sips of the amniotic fluid, moving hands and feet, changing position, and becoming calm, were shown and reviewed as examples of exploratory or attachment related behaviors. In addition, sensitive parenting was discussed. The intervener suggested that even before birth, a parent can “read” his baby's cues (i.e., feeling whether the baby is calm or moving), and act accordingly. In light of this session's theme, “sensitivity chains”, a fragment was shown where Liam spoke gently toward his calm baby. The intervener highlighted this behavior, and commented on the baby being able to continue her sleep or rest. Finally, the importance of mutual enjoyment and playing together was emphasized.

### 4.3 Evaluation

#### 4.3.1 Intervener's experience

The intervener described Liam as enthusiastic, yet somewhat reserved. Liam showed great interest in the ultrasound images, and it seemed as if he therefore was not always able to listen to the intervener's live feedback when provided. Liam appeared to experience difficulties with performing certain tasks, which possibly distracted him from making contact with his baby. With time, however, Liam seemed more comfortable, cooperative, and open to the intervener's feedback. He actively participated in the sessions, remembered the feedback that was provided at an earlier stage, and posed questions. With regard to the first profile item (understanding of what moves the baby and showing empathy), Liam regularly spoke directly towards the baby and seemingly got more aware of the fact that his baby has certain experiences and preferences (e.g., “*How did you like the book I read to you?*”). Progress was visible as Liam indicated he felt a stronger emotional bond to the baby than before. Also, realizing that the baby was able to recognize his voice made him read books to her. With regard to the second profile point (noticing of signals and observing the baby), Liam became more aware of the baby's responding to his actions and he indicated this had a great impact on him. This was also evident from fragments where Liam carefully observed his baby on the ultrasound images before starting with a task.

#### 4.3.2 Father's evaluation

Liam reported he had gained more insight in his relationship with the baby, understanding of the baby, and communication with the baby, with scores of 3 (out of 5). He indicated a smaller increase in understanding of his baby's feelings, with a score of 2. Liam considered the intervention sessions as somewhat helpful, and rated the interaction with the intervener as pleasant, with scores of 2 and 4, respectively. Liam's response to the question what he liked most about the sessions was: *“Seeing the baby and her reaction to my voice was special.”* Singing for the baby during one of the sessions was the thing Liam liked least.

#### 4.3.3 Sensitivity and involvement assessments

Liam's parenting sensitivity scores were 3.5 at baseline, 6.5 after completion of the intervention, and 4 post birth. These scores indicated an increase in parenting sensitivity from baseline to the post‐intervention assessment. Although post birth sensitivity was lower than post‐intervention sensitivity, there was still a slight increase visible from baseline to post birth. In addition, Liam scored .51 at baseline, .59 after completion of the intervention, and .89 post birth for involvement with the baby, and .54, .48, and .45 for accessibility, respectively. For involvement, an increase was visible from baseline to the post‐intervention assessment and – even more evident – to post birth. For accessibility, a slight decline was visible from baseline to post birth assessments. Again, caution is needed when interpreting these results given the absence of a control condition in the current study.

## 5 DISCUSSION

VIPP‐PRE is a recently developed prenatal parenting intervention program using live ultrasound images of father‐fetus interactions, aiming to enhance fathers’ postnatal parenting sensitivity and paternal involvement. Here, we provided an overview of the intervention process based on two case studies of expectant fathers receiving VIPP‐PRE. For both cases, the three intervention sessions were described during which father‐fetus interactions were recorded and reviewed. Fathers received live feedback during the recordings, as well as script‐based feedback while reviewing the videos during the next session. Feedback was both standardized, using messages from the VIPP‐PRE manual, and personalized, based on specific fragments and videotaped fetal and paternal behavior. We also reviewed fathers’ and intervener's evaluation, and noted changes in observed levels of prenatal and postnatal sensitivity and involvement.

### 5.1 Ultrasound imaging

Video recordings of parent‐child interaction are a crucial element of the VIPP, showing parents “their child's perspective as well as their own parenting behavior as if they are looking in a mirror” (Juffer & Bakermans‐Kranenburg, 2018, p. 1355). The most evident change of VIPP‐PRE relative to other VIPP programs is the usage of ultrasound imaging for the recording of these videos and the addition of live feedback. The current study showed that fathers were positive about the ultrasound images and the opportunities to see their baby, as well as its behavior and responses. Ultrasound imaging is known to help expectant fathers to see the pregnancy as real and develop an emotional bond to the unborn baby (Poh et al., 2014). Using this method to record real‐life interactions between fathers and their unborn babies is unique in prenatal interventions and guarantees ecological validity. The current study also showed that it is feasible to combine ultrasound images with other elements of the intervention. For example, it was possible to capture and emphasize fragments of positive interactions, which may offer opportunities to enhance feelings of competence and stimulate fathers’ involvement with their baby. Also, video feedback was personalized and “speaking for the baby” techniques were applied in order to work on fathers’ perspective taking, interpretation of child signals, and empathizing. Thus, using ultrasound imaging appears to be a suitable alternative for visualizing and discussing father–child interactions, one that provides unique opportunities for intervening in the prenatal phase. Moreover, the equipment that we used can also be used by the intervener at the couples’ homes, without the need for an ultrasound technician. This greatly advances the program's feasibility with full‐time working fathers or mothers, for whom sessions can be scheduled at home during the evenings.

Yet, the use of ultrasound imaging also entailed challenges. First, due to difficulties with imaging near the end of pregnancy (i.e., with regard to visibility of the fetus), sessions preferably took place before 30 weeks of gestation. As sessions started after a confirmation of a healthy physical fetal development by a standard 20‐week ultrasound scan, the time window was limited and the number of sessions was reduced from six in the original VIPP to three in VIPP‐PRE. As a consequence, the theme “sharing emotions” (e.g., comforting a sad child, sharing joy during play) of the regular intervention program is not included in the VIPP‐PRE program. However, as it is not possible to visualize fetal emotions by means of ultrasound imaging, it can be argued that this theme was the least suited for the prenatal intervention. Second, VIPP‐PRE also included live feedback that was provided while recording the videos, because the ultrasound images of the baby are not necessarily clear to the untrained fathers’ eyes. However, this required fathers to multitask by looking at the ultrasound images and listening to the intervener while doing so. One of our two case study fathers seemed to have a hard time concentrating on the intervener's messages as he was entirely preoccupied with looking at his baby on the screen. As also proposed earlier (Alyousefi‐van Dijk et al., 2021), it would be interesting to further examine the added value of providing live feedback. Third, prenatal administration and using ultrasound imaging requires a more hypothetical approach at some points, with a broader interpretation of attachment‐related behaviors as it is more challenging to visualize fetal behavior than the infant's behavior after birth. However, the method does provide opportunities to discuss the VIPP themes and is an essential part of the prenatal VIPP.

### 5.2 Fathers

Over the past decades, the role of fathers in their children's lives has expanded dramatically, at least in Western, industrialized countries (Alio et al., 2013; Bakermans‐Kranenburg et al., 2019). Unfortunately, this greater investment of fathers in child rearing is not reflected in the current availability of parenting support programs, particularly not in the perinatal phase. At the same time, pregnancy and the first years of life are considered to be of critical importance for a child's further growth and development (Berg, 2016). As fathers still experience barriers to be engaged in these early stages of parenthood (Moss, 2019; Steen et al., 2012; Widarsson et al., 2015), early parenting support may be particularly beneficial for them, boosting their involvement and sensitive behavior and thereby laying a foundation for the years to come.

VIPP‐PRE is the first adaptation of the VIPP programs that has been developed specifically for (expectant) fathers. In the current study, both fathers evaluated the intervention as generally positive. They reported more insight into the relationship with their baby, the communication with their baby, and understanding of their baby after completion of the VIPP‐PRE. In the complete sample of 73 fathers, we also demonstrated that the intervention promoted expectant fathers’ insights into their babies (Alyousefi‐van Dijk et al., 2021). However, fathers reported only slight increases in their understanding of the baby's feelings after the intervention. This may reflect the intervention's difficulties in visualizing facial expression and emotions of the unborn child. In addition, one father found the intervention only somewhat helpful, and reported that he disliked the element of singing during the intervention. It could be that interaction with an unborn infant, or parts of that interaction, are unusual or new for some fathers, particularly when taking place in a lab setting and in the presence of an unknown intervener. Their feeling somewhat uncomfortable may however disappear over time when they continue to interact with their unborn babies at home and come to enjoy it. Having said that, singing may be awkward for some fathers and might better be replaced with book reading or telling a story as alternatives.

One father indicated that he would have preferred involving his partner in the intervention as well. Given the scarcity of prenatal parenting interventions for expectant fathers, VIPP‐PRE focuses specifically on fathers aiming for increasing or boosting their sensitivity and involvement. It should be noted that VIPP‐PRE is particularly suitable for fathers, in contrast to mothers, as they are able to place their heads close to the abdomen when interacting with the fetus. It would be beneficial for future studies to involve mothers more in VIPP‐PRE and also examine couple dynamics during and after VIPP‐PRE. Both fathers did report positively about their experiences with the intervener. This was also similar to findings in the complete sample (Alyousefi‐van Dijk et al., 2021). Establishing a supportive working relationship with the parent is related to more optimal treatment outcomes (Shirk & Karver, 2003) and is therefore, as in other VIPP programs, an essential part of VIPP‐PRE. The overall positive evaluations of the intervention and experiences with the intervener indicate that VIPP‐PRE can be an acceptable intervention for fathers.

### 5.3 Paternal sensitivity and involvement

The positive effects of paternal sensitivity and involvement on child outcomes have been established in multiple studies (e.g., Dubowitz et al., 2001; Lucassen et al., 2011). At the same time, fathers have been found to be less sensitive in parenting (e.g., Fuertes et al., 2016; Hallers‐Haalboom et al., 2017; Volling et al., 2002) and less involved in their children's lives than mothers (McWayne et al., 2008). Fathers may thus profit from parenting interventions. Furthermore, as paternal sensitivity and involvement are known to at least partly originate in the prenatal phase (Cabrera et al., 2008; Hechler et al., 2019; Zvara et al., 2013) this period seems to offer great opportunities for the early enhancement of fathers’ parenting abilities.

In the current case study, assessments of the fathers’ parenting sensitivity and involvement showed some positive changes, independent of their baseline scores on sensitivity. This might indicate that, through stimulating positive father–fetus interactions and providing personalized video feedback (e.g., “speaking for the baby”), VIPP‐PRE facilitates fathers’ recognition and interpretation of child signals, perspective taking, empathy, and his feelings of competence and importance, resulting in increased sensitivity and involvement. However, some increases in sensitivity and involvement were modest, and for one father mixed, with sensitivity decreasing from post intervention to postnatal assessment. Besides, due to the fact that only two cases were included in the current study, we cannot draw firm conclusions about effects on paternal sensitivity and involvement in general. The RCT, of which these two cases were drawn, is necessary to systematically examine the efficacy of VIPP‐PRE on parental sensitivity and involvement. The manuscript describing these results is currently under review.

## 6 CONCLUSION AND IMPLICATIONS

In conclusion, we presented two case studies of expectant fathers receiving VIPP‐PRE in order to support their interaction with their unborn baby, with increased parental sensitivity and involvement after the birth of the baby as the ultimate aim. Our case studies demonstrate positive evaluations, suggesting that VIPP‐PRE, using live ultrasound images, has the potential to enhance fathers’ understanding of their baby. The use of ultrasound imaging in a prenatal parenting intervention may open new ways to support parents in the earliest stages of parenthood. The fact that the current study includes only two cases precludes drawing firm conclusions about effects of VIPP‐PRE on paternal parenting quality. In a RCT, preregistered on https://osf.io/487xc and currently under review, the efficacy of the VIPP‐PRE program is systematically investigated. When proven effective, future research might investigate whether VIPP‐PRE can be beneficial in clinical populations and families at risk.

## CONFLICT OF INTEREST

The authors report no conflict of interest.

## PARTICIPANT CONSENT

All participants and their partners gave written informed consent.

## Data Availability

The data that support the findings of this study are available from the first author, Noor de Waal, upon reasonable request.

## APPENDIX A PROFILE ITEMS

### A.1

1. Noticing of signals, observing the baby,
2. Understanding of what moves the baby, empathy: in your role as intervener you can give information about the development of babies, such as “babies of this age tend to sleep a lot because they need their energy to grow…,”
3. Adequately reacting (including compliments) to positive signals of the baby, such as reactions to father's speech or touch,
4. Noticing and correctly interpreting the behavior of the baby, and reacting correctly to it (doing something about it): in your supportive role you can encourage the correct reaction of the parent,
5. Not interfering or disturbing the baby when he/she is exploring or playing,
6. Adjusting the pace of the parent to that of the baby (usually this will mean: introduce breaks),
7. Good communication between the parent and baby – playful interaction and sharing of feelings,
8. Warmth in the parent's voice and facial expression when interacting with the baby,
9. The “following” of the baby by the parent: looking at what the baby is moving towards, going along with how active the baby is, etc.
